# Auditory-Motor Mapping Training: Comparing the Effects of a Novel Speech Treatment to a Control Treatment for Minimally Verbal Children with Autism

**DOI:** 10.1371/journal.pone.0164930

**Published:** 2016-11-09

**Authors:** Karen Chenausky, Andrea Norton, Helen Tager-Flusberg, Gottfried Schlaug

**Affiliations:** 1 Music and Neuroimaging Laboratory, Department of Neurology, Beth Israel Deaconess Medical Center, Harvard Medical School, Boston, United States of America; 2 Center for Autism Research Excellence, Department of Psychological and Brain Sciences, Boston University, Boston, United States of America; Universidad de Salamanca, SPAIN

## Abstract

This study compared Auditory-Motor Mapping Training (AMMT), an intonation-based treatment for facilitating spoken language in minimally verbal children with autism spectrum disorder (ASD), to a matched control treatment, Speech Repetition Therapy (SRT). 23 minimally verbal children with ASD (20 male, mean age 6;5) received at least 25 sessions of AMMT. Seven (all male) were matched on age and verbal ability to seven participants (five male) who received SRT. Outcome measures were Percent Syllables Approximated, Percent Consonants Correct (of 86), and Percent Vowels Correct (of 61) produced on two sets of 15 bisyllabic stimuli. All subjects were assessed on these measures several times at baseline and after 10, 15, 20, and 25 sessions. The post-25 session assessment timepoint, common to all participants, was compared to Best Baseline performance. Overall, after 25 sessions, AMMT participants increased by 19.4% Syllables Approximated, 13.8% Consonants Correct, and19.1% Vowels Correct, compared to Best Baseline. In the matched AMMT-SRT group, after 25 sessions, AMMT participants produced 29.0% more Syllables Approximated (SRT 3.6%);17.9% more Consonants Correct (SRT 0.5); and 17.6% more Vowels Correct (SRT 0.8%). Chi-square tests showed that significantly more AMMT than SRT participants in both the overall and matched groups improved significantly in number of Syllables Approximated per stimulus and number of Consonants Correct per stimulus. Pre-treatment ability to imitate phonemes, but not chronological age or baseline performance on outcome measures, was significantly correlated with amount of improvement after 25 sessions. Intonation-based therapy may offer a promising new interventional approach for teaching spoken language to minimally verbal children with ASD.

## Introduction

Autism spectrum disorder (ASD) is characterized by deficits in social communication and by repetitive behaviors or restricted interests [1]. Approximately 25–30% of children diagnosed with ASD remain minimally verbal past the age of 5 years [2–4]. Lack of spoken language is associated with severely restricted independence [5, 6] and with elevated rates of self-injurious behavior, aggression, and property destruction [7, 8]. Thus, it is critical for minimally verbal children with ASD to acquire at least some functional words.

Interventions shown to have some efficacy in facilitating the development of functional spoken language in minimally verbal children with ASD include various forms of Discrete Trial Training [9, 10], such as Verbal Behavior [11], Pivotal Response Training [12], and Rapid Motor Imitation Antecedent Training [13]. Other effective interventions, such as the Early Start Denver Model [14], Milieu Communication Training [13] and PROMPT [14], have taken naturalistic or developmental approaches to spoken language development [15]. For a concise overview of the topic, see [16].

Two features are common to the therapies and interventions mentioned above. First, these therapies involve having children imitate spoken words. Second, outcome measures have primarily been based on communication rate (i.e., imitations or spontaneous words per unit time) or on standardized measures of expressive language or vocabulary (e.g., Mullen Scales of Early Learning (MSEL; [17]) or MacArthur-Bates Communication Development Inventory (CDI; [18]).

In the studies above, utterances were judged correct if they were exact or approximate imitations of the target word. Unfortunately, clear descriptions of what qualifies as an approximation are either missing or minimal. Some researchers counted production of only the initial consonant as an acceptable approximation of a word [11]. Others required at least one phoneme (consonant or vowel) of the model to be present in the child’s approximation [19]. Yoder and Stone [20] defined intelligible word approximations as containing “at least one accurate consonant and vowel combination occurring in the correct position and… either the correct number of syllables or a developmentally appropriate syllable reduction” (p. 704); however, rates of interobserver agreement are not provided. While independent use of words in functional contexts is an important skill for minimally verbal children with ASD, it is equally important to address speech production skill, as increased intelligibility improves the degree to which a child’s conversational partners will understand his/her words [20].

We report here a more comprehensive analysis of treatment effects from the use of Auditory-Motor Mapping Training (AMMT; see our proof-of-concept paper [21]), an intonation-based intervention specifically designed to facilitate the development of spoken language in minimally verbal children with ASD. AMMT involves intoning 2-syllable target words or phrases while simultaneously tapping on electronic drums (tuned to the same two pitches used for intoning targets) in an alternating pattern, thus co-activating shared auditory and motor representations of the same manual and vocal actions [22–24] and recapitulating the developmental relationship between manual and vocal motor actions [25–29]. The use of intonation or music-supported activities to facilitate spoken language development in minimally verbal children with ASD has been described in case reports documenting its utility in teaching individual children to produce single words and word combinations [30, 31] and its neurological basis has been discussed in other work [32–34]. Recently, we reported on the results of a proof-of-concept study [21] supporting a possible effect of AMMT in improving verbal output in six minimally verbal children with ASD ranging in age from 5;9 to 8;9, showing statistically significant improvement in a within-subject analysis over 40 therapy sessions (see also Smith et al., 2007 for recommendations and guidelines with regard to conducting and reporting psychological interventions in minimally verbal forms of autism).

Here, we expand upon our previous work [21], now including 23 minimally verbal participants with autism who were treated with AMMT (excluding three pilot participants who were treated during the development of the therapy; and two subjects, one undergoing AMMT and one SRT, who were observed to speak in sentences during Baseline assessments and thus were determined not to be minimally verbal). These subjects will be reported on elsewhere.

The present study fills a gap in the literature on spoken language therapy for minimally verbal children with ASD by comparing AMMT to a non-intoned control therapy in a group of school-aged children. The matched control condition, Speech Repetition Therapy (SRT), involves imitation and repetition of spoken (not intoned) stimuli, produced at a normal speech rate; and does not involve tapping on drums or bi-manual movement of any kind. It is designed to be similar in these respects to conventional forms of speech therapy, while lacking the intonational elements of AMMT.

In the current study, we examined not only the percentage of syllables approximated but also the percentage of consonants and vowels produced correctly. The aims were, first, to determine whether 25 sessions of AMMT would facilitate improvement in spoken language in school-aged minimally verbal children with ASD and, second, to ascertain whether AMMT would lead to greater improvement than SRT. Specifically, we addressed the following questions:

1. Over 25 therapy sessions, would AMMT result in a statistically significant improvement in percentage of approximately correct syllables and in percentage of consonants and vowels correct?
2. How would AMMT compare to SRT on those outcome measures when participants were matched on chronological age, mental age, and pre-treatment test scores?

## Materials and Methods

### Participants

A pilot phase that included two minimally verbal and one verbal participant with ASD was used to develop, refine, and standardize the AMMT intervention; those children are not discussed here. In a second phase, 10 minimally verbal children between 5 and 9 years of age (seven male), diagnosed with ASD by a pediatric neurologist or neuropsychologist prior to enrollment, underwent 40 sessions of AMMT; one of those 10 had an additional 20 sessions after the 40 sessions (total of 60 sessions) of AMMT. Six of those 10 subjects were reported on previously [21]; four of those 10 subjects were not analyzed at the time of the original manuscript submission. All subjects of this second phase are now reported in this paper. In a third phase, 13 minimally verbal children with ASD (13 male) participated in 25 sessions of AMMT and eight minimally verbal children with ASD (six male) received 25 sessions of SRT. Assignment of participants to AMMT or SRT was interleaved while the SRT children were being enrolled. Approximately twice as many children were enrolled in AMMT than SRT, with the goal of matching SRT children to AMMT participants. Seven of the children who received SRT were matched to seven AMMT-treated children on the basis of chronological age, mental age, and performance on the Kaufman Speech Praxis Test (KSPT; [35]) and a test of phoneme repetition; their performance is discussed below. The eighth participant did not meet criteria for being minimally verbal after Baseline assessment. An additional 30 children with autism were found to be ineligible for this study because they could not participate in table-top activities for at least 15 minutes, were unable to imitate any speech sounds, were completely non-vocal, or had other medical/neurological exclusion criteria. Table 1 details characteristics of the included participants.

**Table 1:** Participant Characteristics.

	CA^1^	MA^2^	KSPT ^3^	Phonemic Inventory^4^(mean ±SD)
		
	(mean, [range])	(mean ±SD)	(mean ±SD)	
**Overall Group:**				
**23 AMMT**	6;5 [3;5–9;8]		19.8 ± 10.6	7.5 ± 4.5
**7 SRT**	5;8 [3;9–8;5]		13.9 ± 4.4	8.9 ± 5.4
**Matched Group:**				
**7 AMMT**	6;1 [3;5–8;11]	20.4 ± 8.1	15.4 ± 10.4	7.1 ± 3.4
**7 SRT**	5;8 [3;9–8;5]	22.3 ± 10.8	13.9 ± 4.4	8.9 ± 5.4

1. CA: chronological age (y; mo).

2. MA: mental age (mo), from the Mullen Scales of Early Learning.

3. KSPT: Kaufman Speech Praxis Test, Sections 1 and 2. Raw scores are reported, as standard scores are uninformative for this population. Maximum score is 74.

4. Phonemic Inventory: the number of English vowels and consonants a child is able to imitate. Maximum is 31 phonemes.

Children were recruited from multiple autism clinics and resource centers serving the Greater Boston area. The study was approved by the Institutional Review Board of Beth Israel Deaconess Medical Center, and parents of all participants gave written informed consent prior to enrollment.

Diagnostic status was confirmed by a Childhood Autism Rating Scale (CARS; [36]) score greater than 30 or an Autism Diagnostic Observation Schedule (ADOS; [37]) score greater than 12. Minimally verbal status, confirmed by parent report and child performance during initial assessments, was defined as using fewer than 20 intelligible words and no productive syntax. Inclusion criteria were the ability to correctly repeat at least two speech sounds, participate in table-top activities for at least 15 minutes at a time, follow one-step commands, and imitate simple gross motor and oral motor movements such as clapping hands and opening mouth. One of two tests was used to determine the number of speech sounds children were able to repeat at baseline: (1) the first two sections of the KSPT, or (2) a phonemic repetition test where children were asked to imitate 21 consonants and 10 vowels of English.

While in the study, children continued with their regular school programs but did not participate in any speech therapy activities or new treatments outside of school. Aside from ASD, participants had no other major neurological conditions (e.g., tuberous sclerosis), motor disabilities (e.g., cerebral palsy), sensory disabilities (e.g., hearing or sight impairment), or genetic disorders (e.g., Down Syndrome) that could potentially explain their minimally verbal state.

### Study Design

#### Baseline and Probe Assessments

The study began with a series of baseline assessments, after which treatment commenced. Probe assessments were performed after the 10^th^ therapy session (P10), every five sessions thereafter (P15, P20, P25, etc.), at 4 weeks post-therapy, and at 8 weeks post-therapy.

Baseline and probe assessments evaluated participants’ ability to repeat two sets of 15 bisyllabic words or phrases, Trained and Untrained. Stimuli were intoned (for AMMT participants) or spoken (for SRT participants). A description of therapy session structure appears below. Trained stimuli were explicitly practiced during the intervention sessions. Untrained stimuli were assessed during baseline and probe sessions but not practiced during treatment; their function was to assess the degree to which improvements on trained phrases generalized to novel stimuli. During baseline and probe sessions, prompts for both sets were administered in the same manner used in therapy (i.e., intoned for AMMT participants and spoken for SRT participants), but without practice or corrective feedback. Trained and Untrained stimuli were intermixed and presented in random order.

In order to establish a stable baseline, a minimum of three complete baseline probes was required before beginning the intervention, but because some children required more than one session to complete each probe, the actual number of baseline sessions per participant varied from three to seven. Therefore, we first verified that no improvement had occurred prior to therapy. This is discussed in greater detail below.

As mentioned, probes were also conducted after the 10^th^ therapy session, after every 5^th^ therapy session thereafter, at approximately 4 weeks post-therapy, and at approximately 8 weeks post-therapy. Because the number of therapy sessions varied between 25 and 40 for the two phases of this research, and because (for family reasons) five AMMT participants did not return for the post 4-week probe session and three did return for the post 8-week probe, in this report we compare Best Baseline performance to performance after the 10^th^, 15^th^, 20^th^, and 25^th^ therapy sessions (P10, P15, P20, and P25). Assessments beyond P25, including post-therapy follow-ups, are not reported on here.

#### Stimuli

Trained and Untrained stimuli consisted of 15 high-frequency bisyllabic words or phrases each (30 items total) pertaining to common objects (“bubbles”), actions (“shoes off”) or people (“mommy”) relevant to children’s activities of daily living. The sets contained similar numbers of vowel types and of early-developing ([m, b, j, n, w, d, p, h]), middle-developing ([t, ŋ, k, g, f, v, tʃ, ʤ]), and late-developing consonants ([ʃ, θ, ð, s, z, l, ʒ, r]) [38].

#### Treatment Session Structure

Words in AMMT trials were intoned on two pitches that follow the words’ natural prosodic contour, at a rate of one syllable per second. Target words/phrases were accompanied by simultaneous tapping on electronic drums tuned to the same two pitches (Middle C, 261.6 Hz; and E♭, 311.1 Hz), one tap per syllable. A straightforward relationship between musical notes and prosodic structure was chosen because music and language understanding are related to the level of language disorder [39]. Words in SRT trials were spoken (not intoned) at a normal speech rate, and drums were not included. Aside from these differences, the structure of both AMMT and SRT sessions consisted of the steps described below:

1. **Listening:** Therapist introduces target phrase by showing a picture and using it in a semantic context: “When you were little, you were a *baby*.” Therapist produces target.
2. **Unison:** “Let’s say it together: ‘*baby’*.” Therapist produces target with the child.
3. **Unison fade:** “Again: ‘*ba*..*’*.” Therapist produces initial portion of the target with child, then fades out while child continues on his/her own.
4. **Imitation: (4a)** “My turn: ‘*baby’*.” Therapist produces phrase alone. **(4b)** “Your turn: …” Therapist remains silent while child imitates target.
5. **Cloze:** “Last time: when you were little, you were a…” Therapist presents the same semantic context for phrase; child fills in the blank by producing the target independently.

Treatment sessions took place five days per week, lasted approximately 45 minutes, and included repetition and practice of each of the steps above for the 15 Trained words/phrases. Breaks were provided, during which the child was allowed to play with a preferred toy, have a small snack, or engage in gross-motor activities such as jumping. These occurred after every five to ten items, based on the child’s stamina.

#### Transcription Reliability

All baseline and probe sessions (257 total) were phonetically transcribed and scored by coders blind to the study time point. Each child’s Best Baseline probe (i.e., the one with the largest number of syllables approximately correct, summed over Trained and Untrained stimuli) was identified for comparison with his/her subsequent probe sessions. 10% of probes across participants were transcribed and coded by two independent investigators to assess inter-rater reliability. Results yielded a Cohen’s κ = .497, p < .0005, and 68.0% agreement on syllables approximately correct. For consonants correct, κ = .547, p < .0005, and 70.1% agreement. Finally, for vowels correct, κ = .270, p < .0005, and 54.7% agreement. The values of κ are somewhat lower in this study than has been previously reported for a subset of the participants [21]; this is due to the use of a narrower transcription rubric, designed to identify phonemes absolutely correct as well as syllables approximately correct (see “Measures of Speech Production” below). In addition, values of κ are reduced when the population under investigation is highly unbalanced in its proportion of “correct” and “incorrect” items, while percent agreement is not [40]. Percent agreement rates are commensurate with previously published figures on infant babbles of 76.8% for consonants and 44.8% for vowels [41].

#### Treatment Fidelity

To assess fidelity, treatment and probe sessions were videotaped and monitored to assess therapists’ adherence to the protocol. A total of 26 baseline or probe files (11%) were assessed. On all AMMT trials, stimuli were intoned and drums used, and on no SRT trials were stimuli intoned or drums used. Over a total of 4680 trials assessed, 29 (0.6%) had repeated steps and 7 (0.1%) had omitted steps.

#### Measures of Speech Production

Three measures were used to assess children’s performance. The primary outcome measure was a global measure of emerging speech production. *% Syllables Approximated* was the percentage of approximately correct consonant-vowel (CV) syllables that a child produced during a probe. A syllable was considered approximately correct if (a) the consonant produced shared two of three phonetic features (voicing, place of articulation, manner of articulation) with the target and (b) the vowel was within the same class as the target, sharing two features (tongue height and backness, which refer to dorsal/ventral and anterior/posterior position within the mouth, respectively) with the target. For example, the utterance [gugi] was considered an approximation of “cookie” ([kʊki]) because the consonants [k] and [g] share place (velar) and manner (stop) features and differ only on voicing ([g] is voiced; [k] is unvoiced). Also, both [u] and [ʊ] are high back vowels, differing only on tenseness ([u], as in “boo”, is tense; [ʊ], as in “book”, is not). The number of approximately correct syllables per probe was divided by the total number of syllables in the stimuli (30 per set; 60 total) to yield % Syllables Approximated.

Two additional secondary outcome measures, new for this analysis, describe articulatory precision and are based on a total of 86 consonants and 61 vowels present in the 30 stimuli. *Percent Consonants Correct* was the percentage of correctly produced consonants and *Percent Vowels Correct* was the percentage of correctly produced vowels.

It is important to clarify that, while previous work (e.g., [11–13]) has counted approximations as correct, here we distinguish between our global measure of speech production, Percent Syllables Approximated, and more stringent measures of consonant and vowel accuracy, Percent Consonants and Vowels Correct. Percent Syllables Approximated is intended as an ecologically valid measure that captures the developing intelligibility of these children, similar to the way that word approximations (like “wawa” for “water”) are sometimes counted as vocabulary items for typically developing toddlers [42]. At the same time, however, it is important to be able to compare the performance of these minimally verbal children with that of typically developing children and with children who have speech sound disorders. A common measure is the percentage of consonants or vowels correct [38, 43]). Thus, we include these measures, where the child’s production must be an exact match to the canonical pronunciation, in order to convey the level of performance of these children in a more absolute sense.

## Results

### Examination of Whether Change Occurred Over Baseline Sessions

To ascertain whether repeated Baseline sessions resulted in therapeutic progress, a repeated measures ANOVA on % Syllables Approximated was performed. Two levels of Time were used as a within-subjects factor (first Baseline vs. last Baseline) and Treatment (AMMT vs. SRT) was a between-subjects factor. There was no significant effect of Time. Participants produced a mean of 20.8% Syllables Approximated (SD 17.2) at first Baseline, compared to 22.6% (SD 19.9) at last Baseline, p = .333, Cohen’s d = .09 (very small). There was no significant effect of Treatment, and no significant Time x Treatment interaction. Thus, despite repeated Baseline sessions, we conclude that no significant change took place before therapy.

### Testing for Equivalence between AMMT Subgroups

As mentioned, there were two phases of AMMT research, with different numbers of therapy sessions during each stage. Thus, we deemed it prudent to ascertain whether performance of the 40-session subgroup (n = 10) differed from that of the 25-session subgroup (n = 13). To answer this question, a repeated-measures ANOVA was performed on % Syllables Approximated with Time (Best Baseline vs P25) as a within-subjects factor and Subgroup (25 vs 40 sessions) as a between-subjects factor. Results showed a significant main effect of Time (F(1,21) = 37.920, p < .0005), but no main effect of Subgroup and no Time x Subgroup interaction. Thus, because the two subgroups were not statistically distinct, they were combined in subsequent analyses.

### Degree of Change in the AMMT Group

#### Percent Syllables Approximated

**Fig 1:**
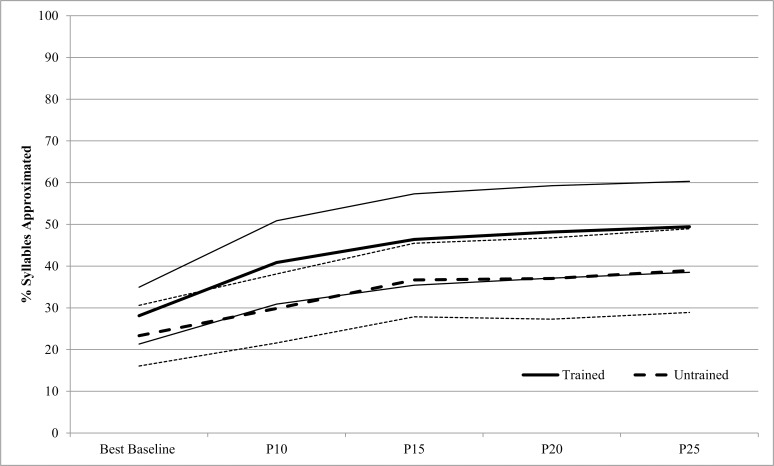
Percent Syllables Approximated By Time and Stimulus Type (AMMT Group). Lighter lines represent 95% confidence intervals.

Fig 1 shows % Syllables Approximated from Best Baseline to P25 for the 23 AMMT participants. A repeated measures ANOVA on arcsine-transformed % Syllables Approximated, with Time (Best Baseline to P25, inclusive) and Stimulus Type (Trained vs. Untrained stimuli) as within-subjects factors, showed a significant main effect of Time, F(4,88) = 14.950, p < .0005. AMMT participants produced a mean of 26.1% (SD 16.5) Syllables Approximated at Best Baseline, compared to 45.5% (SD 25.9) at P25, Cohen’s d = .9 (large). The earliest probe session at which % Syllables Approximated increased significantly over Best Baseline was P15 (p = .007, Bonferroni-corrected). There was also a significant main effect of Stimulus Type, F(1,22) = 23.049, p < .0005. AMMT participants produced a mean of 42.6% (SD 25.9) Syllables Approximated in Trained stimuli, compared to 34.9% (SD 23.3) in Untrained stimuli, Cohen’s d = .3 (small). There was no significant Time x Stimulus Type interaction.

#### Percent Consonants Correct

**Fig 2:**
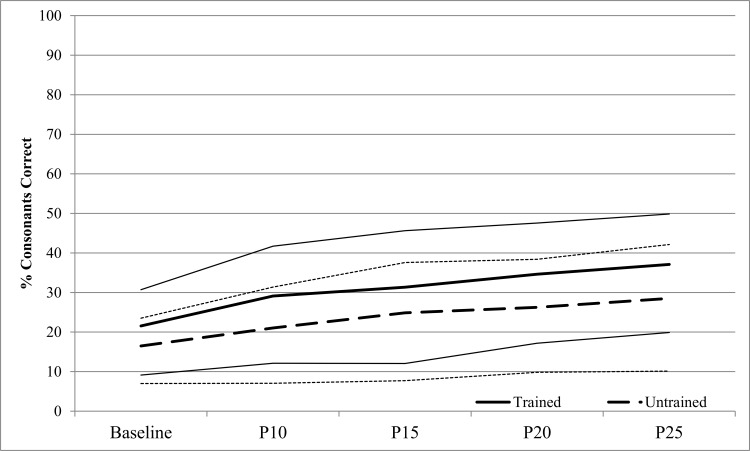
Percent Consonants Correct by Time and Stimulus Type (AMMT Group). Lighter lines represent 95% confidence intervals.

Fig 2 shows % Consonants Correct from Best Baseline to P25 (inclusive) for the 23 AMMT participants. A repeated measures ANOVA with # Consonants Correct as the dependent variable and Time and Stimulus Type as within-subjects factors showed a significant main effect of Time, F(4,88) = 12.777, p < .0005. AMMT participants produced a mean of 19.0% (SD 9.6) Consonants Correct at Best Baseline, vs 32.8% (SD 17.5) at P25, Cohen’s d = 1.2 (large). The earliest probe session at which % Consonants Correct increased significantly over Best Baseline was P15 (p = .047, Bonferroni-corrected). There was also a significant main effect of Stimulus Type, F(1,22) = 15.930, p = .001. AMMT participants produced a mean of 30.7% (SD 17.8) Consonants Correct in Trained stimuli and 23.4% (SD 16.2) in Untrained stimuli, Cohen’s d = .04 (small). There was no significant Time x Stimulus Type interaction.

#### Percent Vowels Correct

**Fig 3:**
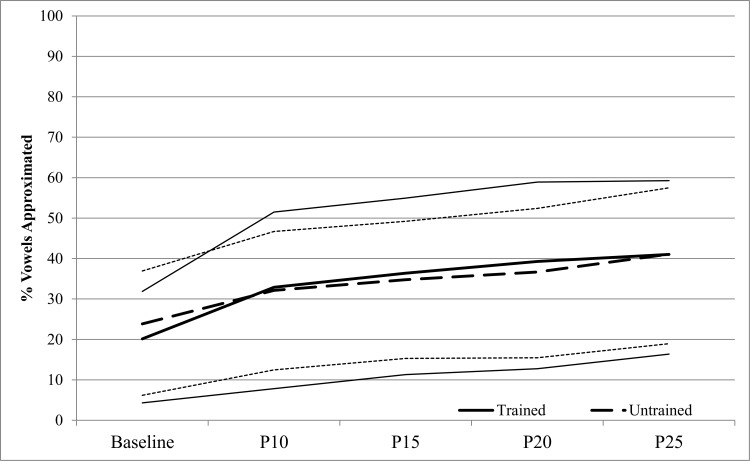
Percent Vowels Correct by Time and Stimulus Type (AMMT Group). Lighter lines represent 95% confidence intervals.

Fig 3 shows % Vowels Correct from Best Baseline to P25 for the 23 AMMT participants. A repeated measures ANOVA with % Vowels Correct as the dependent variable and Time and Stimulus Type as within-subjects factors showed a significant main effect of Time, F(4,88) = 14.456, p < .0005. AMMT participants produced a mean of 22.0% (SD 16.7) Vowels Correct at Best Baseline and 41.1% (SD 23.1) at P25, Cohen’s d = .9 (large). The earliest probe session at which %S Vowels Correct increased significantly over Best Baseline was P10 (p = .007, Bonferroni-corrected). There was no significant main effect of Stimulus Type, and no significant Time x Stimulus Type interaction.

### Comparison of Matched AMMT and SRT Participants

#### Percent Syllables Approximated

**Fig 4:**
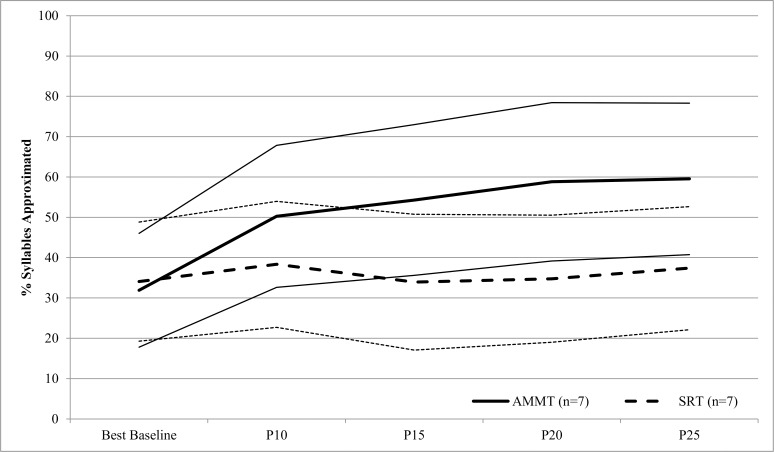
Percent Syllables Approximated by Time and Treatment (Matched Group). Lighter lines represent 95% confidence intervals.

Fig 4 shows % Syllables Approximated from Best Baseline to P25 for the matched AMMT and SRT groups. A repeated measures ANOVA from Best Baseline to P25 inclusive, with Time and Stimulus Type as within-subjects factors and Treatment as a between-subjects factor, was performed on arcsine-transformed % Syllables Approximated. There was a significant main effect of Time, F(4,48) = 12.812, p < .0005. Across both groups, participants produced 32.1% (SD 14.9) Syllables Approximated at Best Baseline, compared to 48.5% (SD 16.9) at P25, Cohen’s d = 1.0 (large). There was also a significant main effect of Stimulus Type, F(1,12) = 14.636, p = .002. Participants produced a mean of 47.9% (SD 16.6) Syllables Approximated in Trained stimuli, vs. 38.4% (SD 15.8) in Untrained stimuli, Cohen’s d = .6 (medium). There was no significant main effect of Treatment, indicating that the groups were not consistently different across all timepoints (specifically, they were equivalent at Baseline and diverged thereafter). Importantly, there was a significant Time x Treatment interaction (F(4,48 = 8.343), p < .0005), indicating that the two groups showed different trajectories during therapy. The AMMT group improved by a mean of 29.0% from Best Baseline to P25; the SRT group by only 3.6% over the same number of sessions, Cohen’s d = 3.0 (very large). There were no other significant two-way interactions and no significant three-way interaction.

#### Percent Consonants Correct

Because visual inspection of the plots of the outcome measures by Time and Treatment showed that the AMMT group’s score on % Consonants Correct was lower at Best Baseline than that of the SRT group, this variable was tested for group differences at Best Baseline to determine whether a correction factor was needed. Independent-samples t-tests on the mean Best Baseline score for AMMT and SRT on % Consonants Correct showed no significant between-group difference (AMMT 16.9% (SD 7.2) vs. SRT 28.9% (SD 17.5), p = .119), although the SRT group had a slightly higher performance than the AMMT group at baseline.

**Fig 5:**
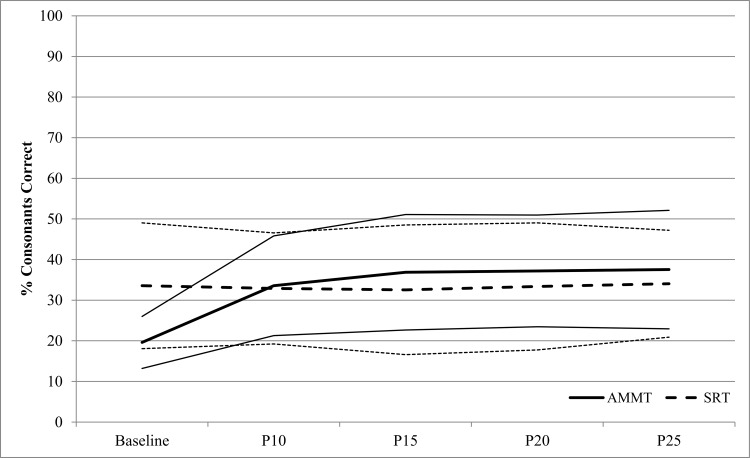
Percent Consonants Correct By Time and Treatment (Matched Group). Lighter lines represent 95% confidence intervals.

A repeated measures ANOVA was then performed with Time and Stimulus Type as within-subject factors and Treatment as a between-subjects factor. There was a significant main effect of Time on % Consonants Correct, F(4,48) = 5.409, p = .001. Participants produced a mean of 26.6% (SD 22.8) Consonants Correct at Best Baseline, compared to 35.8% (SD 27.0) at P25, Cohen’s d = 0.4 (small). There was also a significant main effect of Stimulus Type, F(1,12) = 36.937, p < .0005. Participants produced a mean of 37.2% (SD 25.0) Consonants Correct in Trained stimuli, vs 29.1% (SD 26.5) in Untrained stimuli, Cohen’s d = 0.3 (small). There was no significant main effect of Treatment. However, there was a significant Time x Treatment interaction, F(4,48) = 6.502, p < .0005. AMMT participants improved by a mean of 17.9% Consonants Correct from Best Baseline to P25, while SRT participants improved by only 0.5% Consonants Correct over the same period, Cohen’s d = 1.0 (large). There was also a significant Stimulus Type x Treatment interaction, F(1,12) = 7.537, p = .018. AMMT participants produced a mean of 11.5% more Consonants Correct in Trained than in Untrained stimuli, while SRT participants produced a mean of 4.6% more Consonants Correct in Trained stimuli. There were no other significant two- or three-way interactions. Fig 5 shows % Consonants Correct over time for the matched AMMT and SRT groups.

#### Percent Vowels Correct

**Fig 6:**
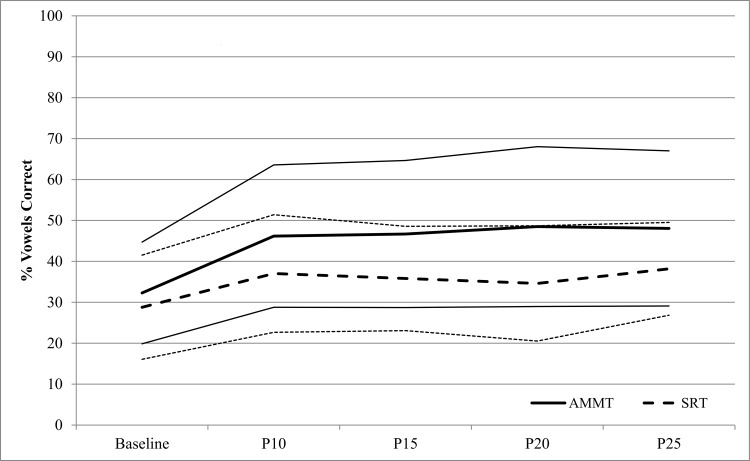
Percent Vowels Correct by Time and Treatment (Matched Group). Lighter lines represent 95% confidence intervals.

Fig 6 shows % Vowels Correct from Best Baseline to P25 for the matched AMMT and SRT groups. A repeated measures ANOVA, with Time and Stimulus Type as within-subjects factors and Treatment as a between-subjects factor, was performed on % Vowels Correct for the Matched participants. There was a significant main effect of Time, F(4,48) = 7.985, p < .0005. Participants produced a mean of 30.6% (SD 16.0) Vowels Correct at Best Baseline, compared to 43.1% (SD 20.5) at P25, Cohen’s d = 0.7 (medium). There were no other significant main effects.

There was a significant Time x Stimulus Type interaction, F(4,48) = 3.893, p = .008. Participants improved by a mean of 15.7% on Trained Stimuli from Best Baseline to P25 (Cohen’s d = 1.1, large), compared to an improvement of 9.5% on Untrained Stimuli from Best Baseline to P25 (Cohen’s d = 0.7, medium). There were no other significant two- or three-way effects.

### Number of Responders Per Group

Though the AMMT group on average showed more improvement than the SRT group on average, it is unrealistic to expect any one therapy to work for all children, particularly for those with ASD. In fact, there were some participants from each group who showed improvement, while others receiving the same treatment did not. To investigate this issue in greater detail and better understand the role of individual differences in response to therapy, we performed an analysis to determine how many participants in each group responded to therapy.

Paired t-tests were used to compare # Syllables Approximated Per Stimulus, # Consonants Correct Per Stimulus, and # Vowels Correct Per Stimulus at Best Baseline and P25 for each participant. For example, the number of syllables approximately correct in each stimulus at Best Baseline was compared with the number of syllables approximately correct in that stimulus at P25, for each child. *Responders* were those participants who experienced a statistically significant increase from Baseline to P25; all others were *Non-Responders*. Chi-square tests for association were then performed on the number of Responders and Non-Responders in each treatment (AMMT vs. SRT), for the Matched group and for the overall group of 23 AMMT and 7 SRT participants.

For the Matched group, there was a statistically significant association between Treatment and # Syllables Approximated Per Stimulus, χ^2^(1) = 10.500, p = .001. For # Consonants Correct Per Stimulus in the Matched group, there was also a statistically significant effect of Treatment, χ^2^(1) = 4.667, *p* = .031. Finally, for # Vowels Correct Per Stimulus in the Matched group, there was no significant effect of Treatment, χ^2^(1) = 1.167, *p* = .280. Results are shown in Table 2.

**Table 2:** Responders (Matched Group).

Outcome Measure	AMMT (n = 7)	SRT (n = 7)
**# Syllables Approximated Per Word**	7/7 (100%)*	1/7 (14%)
**# Consonants Correct Per Word**	5/7 (71%)*	1/7 (14%)
**# Vowels Correct Per Word**	4/7 (57%)	2/7 (29%)

*p < .03

For the overall group of 23 AMMT and 7 SRT participants, there was a statistically significant association between Treatment and # Syllables Approximated Per Stimulus, χ^2^(1) = 11.273, p = .001. For # Consonants Correct Per Stimulus in the overall group, there was also a statistically significant effect of Treatment, χ^2^(1) = 4.658, p = .031. Finally, for # Vowels Correct Per Stimulus in the overall group, there was no significant effect of Treatment. For Syllables Approximated and Consonants Correct Per Stimulus, more AMMT participants than SRT participants showed a significant improvement from Best Baseline to P25. Results are shown in Table 3.

**Table 3:** Responders (Overall Group).

Outcome Measure	AMMT (n = 23)	SRT (n = 7)
**# Syllables Approximated Per Word**	19/23 (83%)*	1/7 (14%)
**# Consonants Correct Per Word**	14/23 (61%)*	1/7 (14%)
**# Vowels Correct Per Word**	15/23 (65%)	2/7 (29%)

*p < .03

#### Relation of Pre-Treatment Factors with Change Scores

As shown in Table 3, a majority of the 23 AMMT participants were Responders to the treatment, according to each outcome measure. To determine what differentiated Responders from Non-Responders, our final analysis focused on the pre-treatment factors that significantly correlated with the change score for each outcome measure. We looked at three factors from each child’s Best Baseline: (1) score on the relevant outcome measure, (2) chronological age, and (3) phonemic inventory (i.e., the number of isolated consonants and vowels a child correctly repeated).

**Fig 7:**
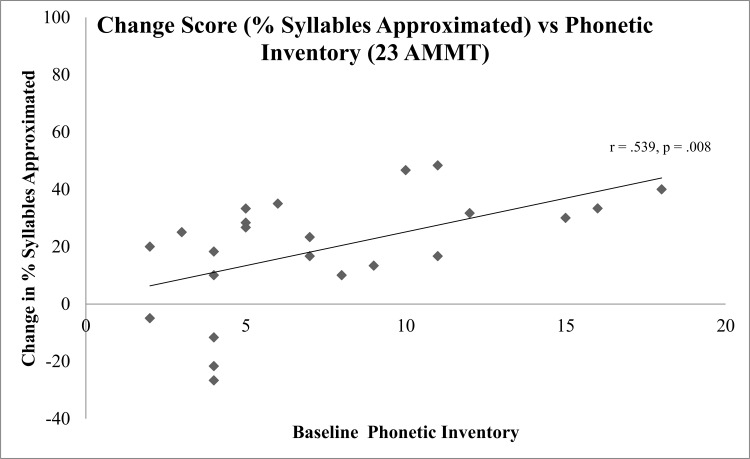
Change Score in Percent Syllables Approximated vs Baseline Change Score.

For no outcome measure was the Best Baseline score on that measure significantly correlated with its respective change score. Neither was chronological age correlated with any change score. However, phonemic inventory score was significantly correlated with the change score in % Consonants Correct (Pearson’s r = 0.430 (moderate), p = .041), the change score in % Vowels Correct (Pearson’s r = 0.419 (moderate), p = 0.047) and with the change score in % Syllables Approximated (r = 0.539 (strong), p = .008). All r-values are shown in Table 4, and the relationship between Change Score in % Syllables Approximated and Phonetic Inventory is shown in Fig 7.

**Table 4:** Correlation between Baseline Scores and Change Scores (23 AMMT).

	Change in % Consonants Correct	Change in % Vowels Correct	Change in % Syllables Approximated
**Baseline Score**^**1**^	.052	-.002	.174
**Chronological Age**	.161	.257	.240
**Phonemic Inventory**	.430*	.419*	.539**

*p < .05

**p = .008

^1^Best Baseline score for % Consonants Correct was correlated with the change score for % Consonants Correct, Best Baseline % Vowels Correct with the change score for % Vowels Correct, and Best Baseline % Syllables Approximated with the change score for % Syllables Approximated.

## Discussion

In this study, we compared two therapies for facilitating spoken language output in minimally verbal school-aged children with ASD. Four main results emerged. First, in the group of 23 AMMT participants, there was a significant improvement in % Syllables Approximated, % Consonants Correct, and % Vowels Correct after 25 therapy sessions. Second, in the matched groups of seven AMMT and seven SRT participants, the AMMT group showed significantly more improvement in % Syllables Approximated and % Consonants Correct; there was no between-group difference in % Vowels Correct. Third, a majority of the 23 AMMT participants responded to treatment on each of the three outcome measures; and significantly more AMMT than SRT participants responded to treatment. Fourth, in the group of 23 AMMT participants, phonemic inventory score at Best Baseline was significantly correlated with the change score in % Syllables Approximated and % Vowels Correct, and the correlation with the change score in % Consonants Correct approached significance. We discuss each finding in turn.

The significant improvement over time on % Syllables Approximated, % Consonants Correct, and % Vowels Correct in both types of stimuli for a group of 23 AMMT participants replicates and extends our previous results in a small proof-of-concept study (Wan et al., 2011). Given the extreme challenges these children face and the challenges of working with them, this demonstration that AMMT can improve spoken language and articulation in minimally verbal children with ASD and that the improvements are associated with large effect sizes represents an important result. The lack of improvement over repeated Baseline assessments (when participants received no corrective feedback), suggests that the gains are associated with therapy, not just exposure to the stimuli. The fact that significant improvement on the outcome measures occurred between Best Baseline and P10 (for % Vowels Correct) and between Best Baseline and P15 (for % Syllables Approximated and % Consonants Correct), with trajectories leveling after P15, shows that the greatest improvement generally occurs within the first 15 therapy sessions.

The lack of a Time x Stimulus Type interaction over the course of therapy indicates that the children in this study were able to effectively generalize the skills they learned in therapy to words they had not practiced. However, the presence of a consistent main effect of Stimulus Type on % Syllables Approximated and % Consonants Correct deserves comment. The stimulus sets were matched for number of early-, middle- and late-developing consonants; however, there were more unvoiced stops in the Untrained stimuli than in the Trained stimuli (ten vs five). The lower performance across the board on the Untrained stimuli may therefore be an effect of phonetic complexity.

The significant Time x Treatment interaction on % Syllables Approximated and % Consonants Correct shows that AMMT resulted in greater improvement than SRT for the matched participants, and that AMMT’s improvements over SRT were associated with large effect sizes. In addition, AMMT resulted in significant improvement over 25 sessions for 57%-100% of matched participants, depending on the measure, while SRT resulted in significant improvement for only one or two participants per measure. For the overall group, AMMT resulted in significant improvement on all three measures for 14 to 19 of 23 participants, as compared to at most two SRT participants. In the matched group, 57%-100% of AMMT participants showed significant improvement. These figures are commensurate with previously reported proportions of participants showing improvement after therapy ([14], 60–80%; [16] 42–50%). Thus, in this group of participants, AMMT produced significantly greater gains in spoken language acquisition in minimally verbal children with ASD than a control therapy, SRT, which does not contain the critical elements of AMMT (i.e., tapping on tuned drums while intoning bisyllabic words/phrases).

There are several possible reasons for AMMT’s better performance than SRT. First of all, many children with ASD enjoy listening to and making music [44–46]. Including enjoyable musical activities may have increased the effectiveness of AMMT and provided more opportunities for learning than would have taken place in a less enjoyable milieu. In addition, the structure of AMMT therapy requires children to tap one of two tuned electronic drums in sync with each syllable they intone. This may have functioned as a reward, again increasing motivation.

Relatedly, music-making activities such as singing words and phrases, bi-manual tapping on tuned drums in a rhythmic manner, listening to musical sounds associated with learned actions, etc. engage an auditory-motor brain network [23–24]. In particular, the frontal segment of the arcuate fasciculus (AF), the inferior frontal gyrus (IFG), is involved in modality-independent sequencing of perceptual stimuli [47] and the mapping of sounds to actions [24], and it is connected with motor plan selection and execution in premotor and motor areas [48–50]. Through these processes, the IFG and the AF play a fundamental role in the feedforward and feedback control of verbal output. Neuroimaging studies have shown that, relative to typically-developing children, individuals with ASD show micro- and macrostructural abnormalities and asymmetry reversals in the AF [51–53]. Children with ASD also show anatomical and functional reversal of the usual left-right asymmetry in the IFG [51, 54–59], often in the presence of reduced inter-hemispheric connectivity [60]. But auditory and motor regions and the link between them can be specifically engaged through music making activities, especially ones that involve the mapping of hand or finger motor activities with sounds or pitched information [24]. In addition, research suggesting that hand and articulatory movements may share neural correlates [22, 61–63] further supports the notion that hand-tapping is critically important for facilitating the coupling of sounds to orofacial and articulatory actions [24]. To the extent that music and spoken language share neural resources [32], then, AMMT may act as a facilitator of spoken language learning in minimally verbal children with ASD.

A final reason for the increased effectiveness of AMMT over SRT concerns the hypothesis that at least some minimally verbal children with ASD experience, along with cognitive and language impairment, childhood apraxia of speech (CAS) [64]. Treatment of CAS involves (1) the use of early-developing words or phrases and (2) directing the child’s attention to the visual, auditory, and somatosensory aspects of those words or phrases [65]. Imitation, unison production, and a slowed production rate all facilitate speech development in children with CAS [66]. AMMT shares these properties with treatments for CAS. Thus, to the extent that minimally verbal children with ASD may also experience some degree of CAS, the combination of task type and hierarchies from CAS treatment and the use of intoned stimuli and bimanual tapping may have a catalyzing effect, producing better spoken language improvement than either one alone. In the words of Paul et al. [13], therapies that focus on speech production and that give children even a small number of words or word approximations “may be enough to ‘turn on’ the speech learning process” in these children. To the extent that oral-motor skills in infants and toddlers with ASD predict later speech fluency [28], explicitly improving speech oral-motor ability may make it easier for minimally verbal children with ASD to benefit from subsequent language- or social communication-based therapies designed to address other aspects of verbal communication.

Lastly, the fact that only phonemic inventory at Baseline was significantly correlated with the change score in % Syllables Approximated, % Consonants Correct, and % Vowels Correct suggests a possible reason for the improvements seen in this study. Several research groups have examined the integrity of the AF in minimally verbal children with developmental disorders. For example, one group [67] used diffusion tensor imaging (DTI) to show that in six participants with congenital bilateral perisylvian syndrome, more severe language phenotypes were associated with absence of the AF bilaterally. Another group, also using DTI, found atypical lateralization of AF in five minimally verbal children with ASD: four of the five showed reversed asymmetry [52]. A final group [68] replicated these results using DTI tractography to show that in 37 participants with epilepsy and developmental cortical malformations, all participants who lacked a left AF were significantly language-impaired; and those who also lacked a right AF were extremely likely to be nonverbal. The AF is known to support repetition ability in adults [69] and phonological awareness in children [70]. The ability to correctly repeat phonemes may be related to the presence or integrity of the AF in at least one hemisphere. Children with even a small AF in one hemisphere may therefore possess enough of a neural substrate for neural plasticity to take effect and facilitate speech improvement, whereas children who do not demonstrate improvement in speech through therapy may simply lack the neural substrate to develop this skill.

### Clinical Implications

The results reported on here have important clinical implications for the treatment of minimally verbal children with ASD. First, they show that intonation-based therapies can lead to significant improvement in the ability of many of these children to produce some degree of spoken language. While the post-therapy performance of the participants in our study compares favorably to figures for children with speech delay [71], it is important to understand the factors that affect clinical interpretation of these results. The 23 AMMT participants in our study ranged from 3;5 to 9;8 (mean 6;5). On average, after 25 therapy sessions, they produced 45.5% Syllables Approximated (range 5.0–93.4), 32.8% Consonants Correct (range 10.5-67.4), and 41.1% Vowels Correct (range 13.1-82.0) The 54 participants described in [71] ranged in age from 3;6-6;1 (mean 4;3). At entry into their study, their participants produced an average of 66.6% Consonants Correct and 91.3% Vowels Correct. Finally, while the participants in our study produced imitated stimuli, those in [71] produced conversational speech samples of at least 100 utterances. The older age of the participants in our study, and the fact that their productions were imitations rather than spontaneous, should inform the degree and nature of improvement in these children. They experience unusually severe difficulty in producing even imitated speech and have received speech therapy at least since the age of diagnosis. For them to achieve approximately half the accuracy rate in percent consonants and vowels correct that the children in [71] displayed before treatment represents a tremendous amount of hard work on their part.

The amount of improvement in the 23 AMMT participants over 25 sessions of therapy was significantly correlated with the ability to correctly repeat phonemes at Baseline, suggesting that the children who will benefit most from AMMT are those who possess at least a latent ability to imitate speech sounds. Intonation-based treatments thus appear able to harness this ability. Analyses of post-treatment maintenance of the gains documented here are ongoing and will reveal the extent to which participants were able to continue to use the skills they developed, four and eight weeks post-treatment.

Though measures of joint attention, language comprehension, and communicative rate were not collected from these participants, important gains beyond the ability to simply approximate the pronunciation of the stimuli were noted. Therapists noted that children’s ability to attend to the clinician, participate in turn-taking activities (such as rolling a ball back and forth during breaks), and make specific requests (for, e.g., snacks, sensory input such as squeezes, favorite motivators, bathroom breaks) improved over the course of treatment. In addition, many children learned to associate the target words with their respective pictures, often spontaneously naming them during Step 1. Finally, both parents and clinicians noted an increase in speechlike vocalization on the part of many of the children. That is, participants engaged in more vocal play, an important precursor to speech development in typically developing children [72]. Thus, AMMT may offer additional benefit in the areas of social communication and receptive language, help “jumpstart” the speech development process in some minimally verbal children with ASD, and further improve their ability to intentionally vocalize. By providing such opportunities for meaningful vocalization in the context of the therapy sessions, AMMT may also help prepare children for future therapies that include social- and language-oriented goals, as well as in naturalistic interactions with potential communication partners.

### Limitations and Future Research

Because conclusions from this study are limited by the small number of control participants, we are in the process of replicating our results in larger-scale randomized studies. Extending AMMT for use with older minimally verbal children and teens and adapting the treatment to facilitate vocal play and intentional vocalizations in minimally verbal children who are not yet able to imitate speech sounds are both possible next steps. Furthermore, since most of the improvement observed in this study occurred before the P15 assessment, future work should also investigate the efficacy of a shorter course of therapy. If a similar amounts of improvement can be generated in 15 sessions rather than in 25 or 40, therapeutic efficiency would be increased, and participants would be available to take advantage of subsequent therapies that build on the skills they acquired from AMMT. Finally, because no one therapy works equally well for all children with ASD, work is ongoing to identify more predictors of therapeutic progress for each type of therapy. In this regard, further research concerning the presence of signs of CAS in minimally verbal children with ASD could yield important prognostic indicators and illuminate the nature of the challenges these children experience in acquiring spoken language.
